# Health-Related Quality of Life and Functional Status Following Intensive Neurorehabilitation in a Patient after Severe Head Injury with Spinal Epidural Hematoma: A Case Report

**DOI:** 10.3390/jcm12082984

**Published:** 2023-04-20

**Authors:** Jan Gnus, Adam Druszcz, Maciej Miś, Luba Ślósarz

**Affiliations:** 1 Department of Physiotherapy, Wroclaw Medical University, 50-368 Wroclaw, Poland; 2 Research and Development Center, Regional Specialist Hospital, 51-124 Wroclaw, Poland; 3 Department of Neurosurgery, Provincial Specialist Hospital in Legnica, 59-220 Legnica, Poland; 4 Department of Neurosurgery, Health Clinic “Medic” in Walbrzych, 58-306 Walbrzych, Poland; 5 Department of Humanities and Social Science, Wroclaw Medical University, 50-368 Wroclaw, Poland

**Keywords:** health-related quality of life, functional status, spinal epidural hematoma, head injury, neurological deficit, neurorehabilitation

## Abstract

Spinal epidural hematoma (SEH) is a very rare condition associated with trauma or occurring as a complication of lumbar puncture and can appear spontaneously. It manifests with acute pain and neurological deficits, leading to severe and permanent complications. This study aimed to assess changes in health-related quality of life and functional status following long-term intensive neurorehabilitation in a patient after severe sport-related head injury with a related SEH. The 60-year-old male patient experienced bilateral weakness of lower limbs, loss of sensation, and sphincter dysfunction. A laminectomy was performed, followed by a slight superficial and deep sensation improvement. The patient underwent intensive neurological rehabilitation treatment. The proprioceptive neuromuscular facilitation (PNF) method, PRAGMA device exercises, and water rehabilitation were provided. The study outcomes were assessed using the validated questionaries World Health Organization Quality-of-Life Scale (WHOQOL-BREF) and Health-Related Quality of Life (HRQOL-14) for health-related quality of life as well as the Functional Independence Measure (FIM) and Health Assessment Questionnaire (HAQ) for functional status. A beneficial clinical improvement was observed following the intensive rehabilitation using PNF techniques, training with a PRAGMA device, and water exercises in the case of SEH. The patient’s physical condition significantly improved, with an increase in the FIM score from 66 to 122 pts. (by 56 pts.) and in the HAQ score from 43 to 16 pts. (by 27 pts.). Additionally, the QOL level increased after rehabilitation, with an increase in the WHOQOL-BREF from 37 to 74 pts. (by 37 pts.) and a decrease in unhealthy or limited days, as assessed using the HRQOL-14, from 210 to 168 (by 42 days). In conclusion, the improvement in QOL and functional level in the SEH patient were associated with high-intensity rehabilitation, simultaneous integration of three therapeutic modalities, and committed patient cooperation.

## 1. Introduction

Spinal epidural hematoma (SEH) is a very rare condition with an incidence of 1 in 1,000,000 people [1]. It occurs due to blood extravasation into the space between the dura mater and the bone canal of the spine. As a consequence of increasing pressure on the spinal cord, patients experience acute pain and significant neurological deficits [2]; therefore, serious, late complications may occur in each case [1].

Spinal injury, including sports trauma, is the most common cause of SEH [3,4]. In those situations, the hematoma is usually located in the area of the injury, and additionally, it causes ligament tears, increased tension in the traumatic area, and vertebral damage [5]. SEH may also be a complication following a lumbar puncture, antiplatelet therapy in pregnant women, or idiopathic [3,6].

The literature also describes cases of spontaneous SEH with minor trauma during back injury and completely idiopathic SEH without even a minor injury or other mechanical cause [7]. However, such cases are very rare; therefore, they provide a valuable source of knowledge for practitioners of neurology, neurosurgery, and neurorehabilitation dealing with such patients in their everyday practice.

It should also be mentioned that among all traumatic SEH cases, the majority are related to spine fractures, while a minority are associated with high-energy trauma [8]. Additionally, delayed traumatic SEH leading to spinal cord compression can be associated with spontaneous spinal fractures [9]. Moreover, SEH is also reported as a sporadic adverse event after some therapeutic procedures, including manual techniques such as spinal manipulation therapy [10]. Regardless of the leading cause and etiopathogenetic factor, patients with SEH suffer from severe neurological deficits, including lower limb muscle weakness or flaccid paralysis, deep sensation disturbances, and problems with controlling physiological functions, which significantly affects their level of activity and quality of life (QOL). Moreover, so far, there have been no studies focused on the evaluation of the QOL of patients with SEH who underwent comprehensive neurological rehabilitation.

It should be mentioned that our previous paper [11] described a detailed clinical picture of this patient along with a literature review of the topic discussed, without aspects of comprehensive and intensive neurorehabilitation procedures. This study aimed to assess levels of health-related QOL and functional status following long-term intensive neurorehabilitation in a rare case of the patient after severe sport-related head injury with adverse spinal epidural hematoma. We described the long-term and intensive rehabilitation process of a 60-year-old man after a sports head injury and SEH in whom the symptoms developed many hours after the head injury in a site not affected by the injury. First, the patient underwent urgent neurosurgery (laminectomy). Then, three rehabilitation methods were implemented: proprioceptive neuromuscular facilitation (PNF), physical therapy using the PRAGMA device, and water rehabilitation. 

## 2. Case Report

### 2.1. Clinical Description

The research project was approved by the Bioethics Committee of the Wroclaw Medical University (approval no. KB-789/2022). The patient gave his written informed consent after a thorough explanation of the procedures involved, and their anonymity was preserved. The study was carried out in accordance with the guidelines of the Declaration of Helsinki and Good Clinical Practice. This article follows the CARE guidelines for writing clinical cases. 

A 60-year-old male patient suffering from ischemic heart disease with a history of a complete myocardial infarction and nephrolithiasis, who was taking acetylsalicylic acid regularly in a dose of 75 mg daily, presented to the Hospital Emergency Department due to sudden, severe pain in the lumbar region for more than 30 min. More than two days (54 h) earlier, during recreational skiing, he suffered a head injury with a bruise on the right eyebrow and right shoulder due to a push by another skier and a fall to the right side of his body. The patient denied any loss of consciousness. The patient was a physician, so he secured the wound with a dressing and bandage in the right frontal area. The patient experienced headaches, dizziness, nausea, and deterioration of well-being, which spontaneously disappeared three hours after the onset of the head injury. His well-being improved, and he continued regular physical activity and normal daily living. Then, two days later, severe pain in the lumbar region appeared; thus, a medical consultation was necessary.

On admission, the physical examination revealed bilateral pain in the lumbar region, and the Goldflam symptom was difficult to assess. The neurological examination assessed bilateral muscle weakness in the lower limbs at 4/5 based on the Medical Research Council (MRC) scale with the preservation of sensory functions and sphincters. Due to the severe pain, the patient was given metamizole and drotaverine by drip infusion while awaiting the results of laboratory and imaging tests.

After absorbing the infusion, the patient suddenly developed laxity in the lower limbs. Muscle strength was assessed as 0/5 on the MRC scale in both legs. Loss of sense of touch, pain, and temperature, as well as sphincter dysfunction, were also demonstrated.

Laboratory tests, including the clotting system, were normal. Computed tomography (CT) examination of the thoracic and lumbar spine did not reveal any vertebral displacements or bone fractures. Magnetic resonance imaging (MRI) of the thoracic and lumbar spine at the Th9-L1 level revealed an SEH. The signal from the end of the spinal cord was elevated, suggesting spinal cord edema. Due to MRI tests and the patient’s condition, he was qualified for surgical intervention. A laminectomy was performed 48 h after the onset of injury at the level of Th10-Th12 with the removal of the hematoma and decompression of the spinal cord. After surgery, there was a slight improvement in superficial and deep sensation, with no improvement in the motor function of the lower limbs (MRC 0/5). Then, the patient underwent intensive rehabilitation. The MRI image after surgery revealed the state after laminectomy and reduction in the hematoma size, pressing on the spinal cord.

Fifty-four hours after the injury, the neurological examination was performed and revealed: The non-walking patient did not lift the lower limbs off the ground with preserved slight mobility of the toes with low muscle tension. No knee or ankle reflexes were induced. Muscle strength was rated as 0 on the Lovett scale in the lower limbs. In addition, the sensation of pain, touch, temperature, and a feeling deep below the Th10 level was suppressed. Urinary and fecal incontinence was also found.

### 2.2. Intensive Neurorehabilitation

An early neurorehabilitation procedure was started at the rehabilitation department three days after laminectomy surgery. The patient underwent passive and active exercises comprising PNF techniques, training with the PRAGMA device, and water exercises. First, the PNF method was used, and the sessions were conducted six times weekly. After four and a half months of exercise, the muscular strength of the limbs and overall efficiency improved; furthermore, greater activity in locomotion and daily activities was achieved. The convalescent was moved to a wheelchair and continued rehabilitation exercises at home. Five and a half months after surgery, the patient started additional rehabilitation using mechanotherapy with the PRAGMA device, which was conducted three times a week for three hours. In addition, water exercises were introduced in the 11th month from the beginning of rehabilitation.

There is a well-known concept based on the individualization of goals [12] and a shaping approach [13] that significantly improves the chance of obtaining positive results in functional and motor recovery. Our approach to developing patient-specific goals was based on the SMART principle considering Specific, Measurable, Achievable, Relevant, and Time-bound goals as well as assuming individualization of the rehabilitation plan. In the neurorehabilitation of spinal injuries, SMART goals can be used to help patients set and achieve specific goals related to improving their physical function, mobility, and overall quality of life. By using this framework, patients can focus on the areas of their rehabilitation that are most important to them while also tracking their progress and celebrating their achievements along the way. Table 1 shows synthesis of our SMART objectives. 

**Figure 1:**
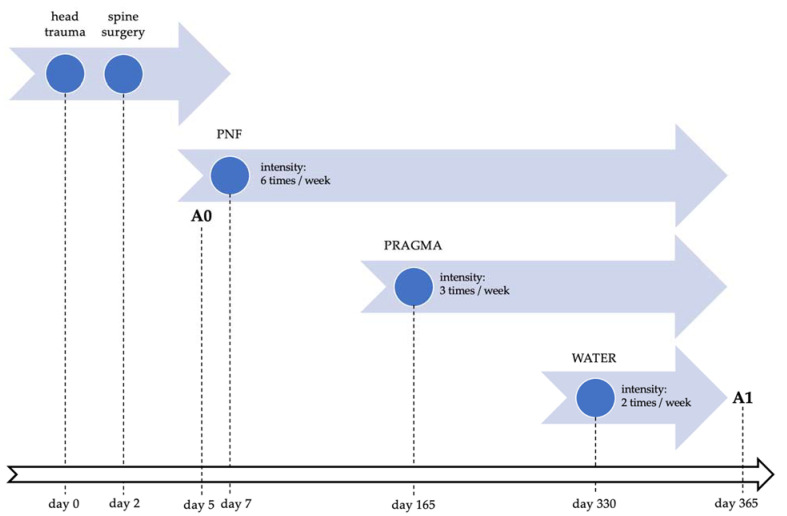
Neurorehabilitation and assessment timeline. Abbreviations: PNF, proprioceptive neuromuscular facilitation; PRAGMA, physical therapy using PRAGMA Pro device; WATER, water rehabilitation; A0, baseline assessment; A1, control assessment.

The patient’s precisely defined needs for achieving a return to the highest possible independence and self-reliance are listed here. Of the greatest importance to the patient was improving his ability to independently assume higher positions and to move and walk with crutches or a walker. Individual rehabilitation sessions based on one-on-one work with a therapist using PNF techniques, as well as task-based training using PRAGMA mechanotherapy and exercises conducted in water, were aimed to re-educate gait patterns and improve activities of daily living. Figure 1 presents a detailed neurorehabilitation and assessment timeline.

#### 2.2.1. PNF Method

PNF was used to improve motor recovery and increase the range of motion. It is a comprehensive and specialist kinesiotherapy. PNF assumptions are based on the neurophysiological principles of movement and the regularities of human motor development. During PNF rehabilitation, the patient is subjected to multisensory stimuli. Resistance, compression, eye, verbal, manual contact, and stretching are used. Resistance is aimed at strengthening muscles, increasing motor control, increasing awareness of movements, and relaxing individual structures. Compression, which consists of traction and approximation, lengthens and shortens the muscle or torso. Eye, verbal, and manual contact, apart from showing the patient the direction and course of movement, gives him a sense of security and stability. Stretching removes increased muscle tension and affects the length of the muscle, which increases flexibility and range of mobility in the joints. PNF techniques are divided into agonistic and antagonistic, depending on the muscles they act on. Agonistic techniques affect only the group of muscles performing the movement. Antagonist techniques work on both the muscle group moving and the opposing muscles. PNF-described techniques include rhythmic initiation, a combination of isotonic contractions, reversal of antagonists, dynamic reversal of antagonists, stabilizing reversal, rhythmic stabilization, repeated stretch, repeated stretch from the beginning of the range, and repeated stretch through a range [14]. 

Subsequent and adequate PNF techniques aimed at functional goals were used in the described patient. One of the main goals of PNF therapy was to work with the patient on a mat in individually selected starting positions (mostly lower). We worked on the mat to ensure a safe position change in body alignment and a larger support plane during therapy to allow the use of global movements. Work was carried out on exercising position changes and moving to higher positions from lying prone and supine. The PNF method was used for early re-education of the individual gait phases in low-lying positions by restoring correct muscle activation or optimal joint angular position. Gait facilitating considered the involvement of the trunk, during which much attention was paid to achieving a stable trunk. Activities were also focused on proper pelvic alignment and mobility of the lower limb joints according to the principle of “mobility on stability.” Work has also been performed to improve the efficiency of the lower trunk muscles, prevent contractures, and improve control and precision of movement, proprioception, and balance control. This was achieved using pelvic patterns in dynamic movement stimulation techniques and combinations of isotonic contractions (concentric, eccentric, and isometric). In the case of lower limb contractures, the “hold-relax”, “tighten-relax,” or “hold-relax-tighten” techniques worked well. It should be noted that a combination of PNF techniques, considering their intensity, speed of movements, body position, and engagement, was adopted according to the individual patient’s condition.

#### 2.2.2. PRAGMA Device

PRAGMA PRO device (Pragma System USA Ltd., Bloomington, IN, USA; Minneapolis, MN, USA) is dedicated to mechanotherapy, being used in rehabilitation, and is especially effective for gait re-education in neurological patients. The essence of the device is the use of elastomers in a flexible suspension. This allows the patient to perform the movements in their natural paths and adapt to the current possibilities. The elastomer has features similar to human muscles, such as elasticity and extensibility, and its structure resembles a chaotic tangle of long and thin particles, and it accumulates resting potential elastic energy. It allows the user to perform an auxotonic contraction, which is the most physiological and is used during locomotor and manual activities. Exercises with elastomers can help patients develop motor skills such as strength, speed, endurance, agility, and motor coordination. The PRAGMA device makes it possible to modify the therapy. The range of motion can be changed as needed. It is possible to use an unlimited number of exercises in each axis, plane, and position [15]. 

The PRAGMA device in the presented patient was used in an outpatient center with the support of a professional and certified physiotherapist. This machine provides flexible suspension, which has been used during patient verticalization and gait re-education and when performing exercises in positions that are difficult to reach (four-legged) or impossible to reach for the patient (vertical). Neurorehabilitation of the present patient, assisted by the PRAGMA device, took place in the supine, sitting, and standing positions. The device was also combined with a verticalization bed, a treadmill, and other equipment for gait re-education.

#### 2.2.3. Water Rehabilitation

Due to its properties, water is a perfect environment for rehabilitation and can be used for medical purposes. Water rehabilitation is a valuable method for enhancing balance and mobility among patients with spinal cord injuries (SCIs) [16]. The buoyancy force causes a perceived loss of body weight, reduces joint strain, and reduces musculoskeletal injury risk. A patient in water can move more efficiently, which also affects his well-being. Moreover, hydrostatic pressure exerts pressure on the patient’s body, relaxing the muscles, facilitating exhalation, making it challenging to inhale, and reducing swelling [17]. Another factor is the water resistance the patient has to overcome by moving. It is used to strengthen weakened muscles. This resistance is greater as the body moves faster and with a larger surface area. It can be easily adjusted using fins or boards and increasing the speed of the exercises [18]. The pool exercise sessions lasted 20–30 min and were very well tolerated by the patient. 

The water environment provided stress relief and reduced gravitational load, which facilitated movement guidance and re-education of movement patterns, especially in the weakened muscles of the lower limbs. In turn, the resistance induced by the water provided an additional element of resistance training for the stronger muscle groups of the trunk and upper limbs. In the water, the focus was on increasing joint mobility, reducing muscle contractures, strengthening the weakened paraplegic muscles, reducing muscle atrophy, and improving the overall condition. In addition, the patient declared that the water environment had a very good relaxing and pain-relieving effect.

### 2.3. Rehabilitation Effects

As a result of long, intensive rehabilitation, the patient regained control over sphincter function after six months. After nine months, the patient made the first movements with his lower limbs. After 18 months of rehabilitation, the motor function in the lower limbs improved on both sides to a grade of 4+/5 on the MRC scale, restoring the full range of sensory functions. After 36 months of rehabilitation, a significant improvement in the motor functions of the lower extremities was achieved. However, disturbances in the strength of the biceps femoris and quadriceps femoris muscles remain. This remarkable rehabilitation success is evident in a significant increase in the patient’s independence level in self-service and locomotion. The patient moves without the help of third parties, assisted by a walker and orthopedic crutches, and has actively returned to his profession as a doctor.

#### 2.3.1. Functional Recovery

The Functional Independence Measure (FIM) is a tool designed to describe the state of functional independence of patients with various neurological conditions, including SCIs [19]. Functional efficiency in self-service, sphincter control, mobility, locomotion independence, communication, and social awareness are assessed. For each task, the patient can obtain from 1 to 7 points, where 1 point indicates complete dependence on third parties and 7 points indicates complete independence during performed activities [20]. The FIM was used to assess the effects of long-term and intensive rehabilitation. 

In the present case, the FIM scale results almost doubled after the rehabilitation process (by 56 pts.) from 66 pts. on admission to 122 pts. after rehabilitation. The most significant improvements (+6 pts.) were observed in such activities as bathing; dressing—lower body; toileting; bowel control; transferring into a bed, a chair, and a wheelchair; taking a tub and a shower; walking; and moving in a wheelchair. Detailed results are presented in Table 2.

#### 2.3.2. Disability Level

The Health Assessment Questionnaire (HAQ) is one of the most frequently used instruments to assess patient-reported outcomes regarding their daily activities and limitations, indicating their level of disability. It consists of 20 questions divided into eight sections: dressing, arising, eating, walking, hygiene, reach, grip, and activities. There are two or three questions for each section and four responses to choose from: without any difficulty—0; with some difficulty—1; with much difficulty—2; and unable to do—3. The final score is the sum of the scores from 0 to 24 divided by eight sections, resulting in a range of 0–3. The higher the HAQ score, the greater the disability level [21].

In our study, the total HAQ score decreased from 43 to 16 pts. (by 27 pts.) which indicates a higher level of activity and lower disability. The highest improvements (−2 pts.) were observed in such tasks as rising (chair), eating (lifting), hygiene (washing and drying, bathing, toileting), and activities (shopping, driving). Detailed results are presented in Table 3.

#### 2.3.3. Quality of Life

The abbreviated World Health Organization Quality-of-Life Scale (WHOQOL-BREF) is one of the most common and widely used tools for assessing QOL. This self-administered tool comprises 26 questions, of which 2 questions evaluate the general perception of QOL and general health as well as another 24 questions belonging to four different domains: physical health, psychological health, social relationships, and environmental factors. Answers are scored on a 1–5 Likert scale: disagree or not at all—1 and completely agree or extremely—5. The higher the total WHOQOL-BREF score, the better the perception of the QOL [22].

The WHOQOL-BREF score increased after rehabilitation from 37 to 74 pts. (by 37 pts.). The most significant improvements were observed in psychological health (negative feelings), with an improvement of +4 pts., as well as general QOL and physical health (work capacity), with an improvement of +3 pts. It should be noted that only one domain worsened (−1 pts.) after rehabilitation, namely, psychological health (personal beliefs), while two did not change (0 pts.): social relationships (social support) and environment (health and social care). Detailed results are presented in Table 4.

#### 2.3.4. Health-Related Quality of Life

The Health-Related Quality of Life (HRQOL-14) is a standardized tool comprising three different modules including 14 questions: Healthy Days Core to assess physical and mental health status (4 questions), Activity Limitations Module to assess limitations in daily functioning (5 questions), and Healthy Days Symptoms to assess pain and emotional sphere (5 questions). In addition, unhealthy days represent an estimated total number of days in the past 30 days when the participant felt that his physical or mental health was not good [23].

Improvement was also noted in the HRQOL-14 score, with the number of unhealthy or limited days decreasing from 210 to 168 days (by 42 days) for all tested modules. Activities in the Activity Limitations Module, such as personal care and routine needs, were improved, while there were no differences in terms of limited activities and major impairment, which did not change during the neurorehabilitation period. Detailed results are presented in Table 5.

## 3. Discussion

The primary anatomic structure causing bleeding related to SEH is the internal vertebral venous plexus; less frequently, it is bleeding of arterial origin [24]. The particular sensitivity of this venous plexus is due to the lack of valves and the fact that it creates anastomoses from the base of the skull to the sacrum, making it susceptible to pressure changes in the chest or abdominal cavity.

Besides head trauma, the patient also had other risk factors for SEH, including antiplatelet therapy (75 mg of acetylsalicylic acid daily) and a fall mechanism that resulted in a sudden increase in pressure in the chest. Therefore, in this case, the etiology of SEH appears to be multifactorial, and the exact role head trauma played in the pathogenesis of SEH is unclear. In light of the above-mentioned information, the relationship between head injury and SEH seems unlikely, but it cannot be ignored.

One of the prognostic factors of rehabilitation and recovery after SEH is the duration of decompression laminectomy [25]. In this case, the procedure was performed 66 h after the head injury and 16 h after the SEH was finally diagnosed. A good prognosis is often associated with surgery within 24 h. The delay in performing the laminectomy from the time of injury may have been related not only to the need to transfer the patient to another hospital but also to the delayed hospital admission on the third day following the injury. Another reason may have been the venous nature of the bleeding, which caused acute neurological symptoms (lower limb paralysis, sphincter dysfunction, and impaired touch and temperature sensation) only after the hematoma reservoir was filled with a particular volume of blood and the spinal cord was compressed. In addition, there have been reports in the literature in which appropriate treatment has been delayed not only because of the rarity of SEH (difficulties in clinical diagnosis) but also due to the presence of symptoms similar to other diseases, such as Guillain–Barré syndrome [26].

On the other hand, there are case reports of patients who underwent surgery immediately or relatively quickly after the onset of symptoms (even up to 18 h) for whom rehabilitation was effective, but only slightly, as described Mimata et al. [27]. For this reason, the work of Liu et al. [28], based on a description of 23 cases of SEH and observations, highlights the second prognostic factor of recovery: the degree of preoperative neurological deficit. In the presence of a complete neurological deficit, the prognosis is unfavorable, even if the neurosurgical procedure is performed less than 24 h after diagnosis. In the present case, the patient’s neurological condition was good on admission, but it deteriorated significantly during his stay in the Emergency Department. This fact significantly affected and complicated neurorehabilitation, especially at the initial stage.

PNF as a neurophysiologic method has been well investigated in terms of its clinical efficiency in various neurological conditions [29], including SCI and its consequences [30]. Additionally, rehabilitation training with the PRAGMA device is a new method that might be useful for complications after SEH. In turn, water rehabilitation, also called aquatic therapy, is deemed useful in patients with SCI-related disorders [31].

Another issue is found in comparing the effects of long-term and very intensive rehabilitation on the patient depending on the chosen rehabilitation method. As mentioned above, several procedures were used simultaneously—PNF techniques, training with the PRAGMA device, and water rehabilitation. Considering that all techniques were performed simultaneously and on only one patient, it is impossible to evaluate them individually in terms of their effectiveness.

It should be noted that recent data regarding neurological conditions related to central nervous system injury also indicate that high-dose and intensive neurorehabilitation sessions lead to significant improvement in the locomotor functions in stroke, brain injuries, and SCIs [32]; present favorable clinical outcomes after stroke in areas such as balance, functional state, depression, and QOL [33]; positively affect walk recovery and QOL after stroke [34]; and improve upper limb function, including grip strength and kinematic properties [35].

A question has to be highlighted: What contributed most to the partial motor and functional recovery allowing the patient to return to the medical profession? Based on this experience, the answer is that every minute of the hundreds of hours devoted to intense exercises played a role. In fact, it was their unique combination of activities, hard work, and patience that finally led to rehabilitation success. Additionally, the aspect of motivation is the key to neurorehabilitation. It is well documented that it has a crucial influence on the treatment progress. The individual sense of self-efficacy affects the motivation to perform the appropriate behaviors necessary to achieve the desired rehabilitation outcomes [36,37]. Adequate levels of motivation significantly impact enhancing psychological functioning, increasing self-confidence, and improving the abilities needed to cope with the consequences of a neurological deficit, which in turn promotes a good prognosis in the long term [38,39]. It should be pointed out that the present patient tolerated the rehabilitation treatment well and showed a high level of motivation for the continuation of the therapeutic process.

Based on the presented case study of a patient after SEH, potential practical implications can be proposed for use in similar clinical situations. The time for diagnosis and implementation of appropriate surgical treatment should be kept to a minimum to avoid worsening of the neurological deficit. Making an efficient differential diagnosis and eliminating other causes of the symptoms is also necessary. Rehabilitation management should be individually tailored to the current needs and capabilities of the patient considering the SMART rule and patient-specific goals. Implementation of high-dose and intensive neurorehabilitation oriented toward functional tasks and improvement in daily activities should be considered. A rehabilitation model using a combination of PNF techniques, training with a PRAGMA device, and water exercises may bring about the expected results. An appropriate level of patient motivation, as well as a target-oriented attitude and positive mental mood, are key factors in achieving successful and persistent results.

Practical Implications

Knowledge of an SHE’s clinical picture with all its symptoms enables rapid implementation of the proper treatment and intensive, multi-path rehabilitation, which may help to avoid or reduce the severity of permanent and severe neurological complications, such as paresis. Rehabilitation after SEH is long-term, intensive, and time-consuming, and its outcome is uncertain. The effectiveness of rehabilitation can be predicted based on the time from the onset of symptoms to neurosurgical operation (the milestone is an operation within 24 h), and the degree of preoperative neurological deficit is another determinant of favorable prognosis.

## 4. Conclusions

In the present case study, the effectiveness of rehabilitation was related to its high intensity, the simultaneous integration of three methods, and significant time commitment, combined with the patient’s dedicated cooperation. Such an outstanding result, which attests to the great success of the rehabilitation, was obtained thanks to the patient’s diligent work and the high commitment of the entire medical team. This has led to very successful effects of this long-term rehabilitation. It is impossible to distinguish which method was most effective, but their combination and a high intensity of rehabilitation seem crucial. Further studies should consider the extension of diagnostics and control of the progress of rehabilitation with neuroimaging (MRI) and neurophysiological (EMG) methods.

## Figures and Tables

**Table 1:** The specific SMART goals of our patient during neurorehabilitation process.

**SMART objective 1: bed-to-wheelchair transfers**	
Specific	Achieve independent bed-to-wheelchair transfers to increase mobility and independence
Measurable	Achieve independent bed-to-wheelchair transfers 90% of the time as observed by a therapist
Achievable	Through targeted transfer training and practice, and use of assistive devices as needed
Relevant	Achieving independent bed-to-wheelchair transfers will increase mobility and independence in daily activities
Time-bound	Achieve this goal within 3 months
**SMART objective 2: wheelchair mobility and independence**	
Specific	Improve wheelchair mobility and independence
Measurable	Increase ability to independently navigate the environment in a wheelchair by 50%, as measured by the Wheelchair Skills Test.
Achievable	Through targeted wheelchair skills training and practice
Relevant	Improved wheelchair mobility and independence will increase participation in daily activities and improve quality of life
Time-bound	Achieve this goal within 6 months
**SMART objective 3: sitting tolerance**	
Specific	Increase sitting tolerance to promote participation in daily activities and reduce the risk of pressure sores
Measurable	Increase sitting time by 30 min per week
Achievable	Through gradual and consistent increases in sitting time during therapy sessions and at home
Relevant	Increased sitting tolerance will allow for more participation in daily activities and decrease the risk of pressure sores
Time-bound	Achieve this goal within 2 months
**SMART objective 4: bladder and bowel control**	
Specific	Improve bladder and bowel control to reduce dependence on catheterization and/or medication
Measurable	Reduce the frequency of catheterization or medication use by 50%
Achievable	Through the implementation of a structured bladder and bowel management program
Relevant	Improved bladder and bowel control will increase independence and reduce the risk of complications such as infections
Time-bound	Achieve this goal within 3 months
**SMART objective 5: upper body strength**	
Specific	Improve upper body strength and endurance to facilitate transfers and independent self-care
Measurable	Increase upper body strength and endurance by 25% as measured by the manual muscle test and number of repetitions of upper body exercises
Achievable	Through targeted upper body resistance training and endurance exercises
Relevant	Improved upper body strength and endurance will facilitate transfers and independent self-care, increasing participation in daily activities and improving quality of life
Time-bound	Achieve this goal within 3 months
**SMART objective 6: upper extremity motor function**	
Specific	Improve upper extremity motor function to increase independence in self-care and other daily activities
Measurable	Increase range of motion and strength in the upper extremities by 20%
Achievable	Through targeted physical therapy exercises and functional training
Relevant	Improved upper extremity function will increase independence in self-care and other daily activities
Time-bound	Achieve this goal within 6 months
**SMART objective 7: lower body strength**	
Specific	Improve lower body strength and endurance to facilitate standing and walking with assistive devices
Measurable	Increase lower body strength and endurance by 25% as measured by the manual muscle test and number of repetitions of lower body exercises
Achievable	Through targeted lower body resistance training and endurance exercises
Relevant	Improved lower body strength and endurance will facilitate standing and walking with assistive devices, increasing independence and quality of life
Time-bound	Achieve this goal within 6 months
**SMART objective 8: lower extremity function**	
Specific	Improve lower extremity motor function by increasing muscle strength and range of motion in the legs
Measurable	Increase muscle strength by 20% and increase range of motion by 30% in the legs
Achievable	Through consistent physical therapy exercises and stretching routines
Relevant	Improved lower extremity motor function will increase independence in daily activities and quality of life
Time-bound	Achieve this goal within 6 months
**SMART objective 9: balance and standing stability**	
Specific	Improve balance and standing stability to reduce the risk of falls and increase mobility
Measurable	Improve balance by achieving a score of 70 or above on the Berg Balance Scale
Achievable	Through balance training exercises and proprioceptive training
Relevant	Improved balance and stability will reduce the risk of falls and increase mobility
Time-bound	Achieve this goal within 3 months
**SMART objective 10: walking with a walker**	
Specific	Improve walking with a walker to increase mobility and independence
Measurable	Walk 50 m with a walker without assistance or rest breaks
Achievable	Through targeted gait training exercises, including walking with a walker and use of assistive devices as needed
Relevant	Walking with a walker will increase mobility and independence in daily activities
Time-bound	Achieve this goal within 12 months
**SMART objective 11: gait speed and quality**	
Specific	Improve gait speed and quality to increase mobility and independence
Measurable	Increase gait speed by 25% and improve gait quality to achieve a score of 7 or higher on the Functional Ambulation Category
Achievable	Through gait training, balance and coordination exercises, and the use of assistive devices as needed
Relevant	Improved gait speed and quality will increase mobility and independence in daily activities
Time-bound	Achieve this goal within 6 months
**SMART objective 12: independent living skills**	
Specific	Achieve independent living skills to promote independence in activities of daily living
Measurable	Achieve a score of 80 or above on the Spinal Cord Independence Measure (SCIM)
Achievable	Through targeted occupational therapy interventions and skill-building exercises
Relevant	Achieving independent living skills will promote independence in activities of daily living and improve quality of life
Time-bound	Achieve this goal within 9 months
**SMART objective 13: driving car skills**	
Specific	Regain driving skills to promote independence and community reintegration
Measurable	Achieve a passing score on a driving assessment and obtain a valid driver’s license
Achievable	Through targeted driving rehabilitation interventions and practice sessions
Relevant	Regaining driving skills will promote independence and facilitate community reintegration
Time-bound	Achieve this goal within 12 months
**SMART objective 14: occupational activity**	
Specific	Increase occupational activity to facilitate return to work or vocational pursuits
Measurable	Increase time spent in occupational activities by 20% and achieve a score of 4 or higher on the Craig Handicap Assessment and Reporting Technique (CHART) in the occupation domain
Achievable	Through targeted occupational therapy interventions and graded exposure to work-related tasks
Relevant	Increasing occupational activity will facilitate return to work or vocational pursuits and improve quality of life
Time-bound	Achieve this goal within 12 months

**Table 2:** Functional status assessment using the FIM scale before and after rehabilitation.

FIM Category	Action	A0	A1	Change
Self-care	Eating	7	7	0
	Grooming	7	7	0
	Bathing	1	7	+6
	Dressing—upper	7	7	0
	Dressing—lower	1	7	+6
	Toileting	1	7	+6
Sphincter control	Bladder	1	5	+4
	Bowel	1	7	+6
Transfers	Bed, chair, wheelchair	1	7	+6
	Toilet	1	7	+6
	Tub, shower	1	7	+6
Locomotion	Walk/wheelchair	1	7	+6
	Stairs	1	5	+4
Communication	Comprehension	7	7	0
	Expression	7	7	0
Social cognition	Social interaction	7	7	0
	Problem solving	7	7	0
	Memory	7	7	0
Total score [pts.]		66	122	+56

Abbreviations: FIM, Functional Independence Measure; A0, baseline assessment; A1, control assessment.

**Table 3:** Disability level assessment using the HAQ scale before and after rehabilitation.

HAQ Section	Question	A0	A1	Change
Dressing	Dressing	2	1	−1
	Shampoo	1	0	−1
Arising	Chair	3	1	−2
	Bed	3	2	−1
Eating	Cut	1	0	−1
	Lift	2	0	−2
	Open	1	0	−1
Walking	Walk	3	2	−1
	Climb	3	2	−1
Hygiene	Wash and dry	2	0	−2
	Bath	3	1	−2
	Toilet	3	1	−2
Reach	Reach	1	0	−1
	Bend	3	2	−1
Grip	Open car doors	1	0	−1
	Open jar	1	0	−1
	Turn faucet	0	0	0
Activities	Shop	3	1	−2
	Car	3	1	−2
	Chores	3	2	−1
Total score [pts.]		43	16	−27

Abbreviations: HAQ, Health Assessment Questionnaire; A0, baseline assessment; A1, control assessment.

**Table 4:** Quality-of-life assessment using the WHOQOL-BREF scale before and after rehabilitation.

WHOQOL-BREF Domain	Question	A0	A1	Change
General QOL		1	4	+3
General health		1	3	+2
Physical health	Activities of daily living	1	3	+2
	Medicinal substances *	2	4	+2
	Energy and fatigue	1	3	+2
	Mobility	1	3	+2
	Pain and discomfort	1	3	+2
	Sleep and rest	1	2	+2
	Work capacity	1	3	+3
Psychological health	Bodily image	1	3	+2
	Negative feelings *	1	5	+4
	Positive feelings	1	3	+2
	Self-esteem	1	2	+1
	Personal beliefs	2	1	−1
	Thinking and memory	3	4	+1
Social relationships	Personal relationships	2	3	+1
	Social support	2	2	0
	Sexual activity	1	2	+1
Environmental factors	Financial resources	2	3	+1
	Physical safety and security	1	2	+1
	Health and social care	2	2	0
	Home environment	2	3	+1
	New information and skills	2	3	+1
	Recreation and leisure activities	1	2	+1
	Physical environment	1	3	+2
	Transport	2	3	+1
Total score [pts.]		37	74	+37

Notes: * the score of these WHOQOL-BREF answers was reversed. Abbreviations: WHOQOL-BREF, World Health Organization Quality-of-Life Scale; A0, baseline assessment; A1, control assessment.

**Table 5:** Health-related quality-of-life assessment using HRQOL-14 scale before and after rehabilitation.

HRQOL-14 Module	Question	A0	A1	Change
Healthy Days Core	General health status	poor	poor	none
	Physically unhealthy days	30	22	–8
	Mentally unhealthy days	30	15	–15
	Activity limitation days	30	28	–2
Activity Limitations Module	Limited activities	yes	yes	none
	Major impairment	code 14	code 14	none
	Period of the problem	30 days	12 months	N/A
	Personal care	yes	no	improved
	Routine needs	yes	no	improved
Healthy Days Symptoms	Pain	30	30	0
	Emotions	30	23	–7
	Worry	30	30	0
	Rest	30	20	–10
	Energy	0	0	0
Total score (days)		210	168	–42

Abbreviations: HRQOL-14, Health-Related Quality of Life; A0, baseline assessment; A1, control assessment.

## Data Availability

The data presented in this study are available on request from the corresponding author (J.G.).
